# Data describing IFNγ-mediated viral clearance in an adult mouse model of respiratory syncytial virus (RSV)

**DOI:** 10.1016/j.dib.2017.07.034

**Published:** 2017-07-19

**Authors:** Katherine M. Eichinger, Kerry M. Empey

**Affiliations:** a Department of Pharmacy and Therapeutics, Pittsburgh, PA, USA; b Center for Clinical Pharmaceutical Sciences, University of Pittsburgh School of Pharmacy, Pittsburgh, PA, USA; c Department of Immunology, University of Pittsburgh School of Medicine, Pittsburgh, PA, USA; d Clinical and Translational Science Institute, University of Pittsburgh, Pittsburgh, PA, USA

**Keywords:** Immunology, Cytokine, RSV, IFNγ, Mice

## Abstract

The data presented here are related to the research article entitled “Age predicts cytokine kinetics and innate immune cell activation following intranasal delivery of IFNγ and GM-CSF in a mouse model of RSV infection” (Eichinger et al., 2017) [1]. The cited manuscript demonstrated that the macrophage-stimulating cytokine, interferon gamma (IFNγ), but not granulocyte macrophage-colony stimulating factor (GM-CSF), effectively enhanced viral clearance in infant mice infected with respiratory syncytial virus (RSV) following intranasal delivery. This article describes the immune response and viral clearing effects of intranasal IFNγ in RSV-infected adult BALB/c mice demonstrating delayed production of endogenous IFNγ. The dataset is made publicly available to extrapolate the role of IFNγ in RSV-infected adult mice.

**Specifications Table**

**Table:** 

Subject area	*Immunology, virology*
More specific subject area	*Effects of pulmonary cytokines on infant RSV infection and immunity*
Type of data	*Text file and graphs*
How data was acquired	*Flow cytometry (LSR II), Luminex (BioRad)*
Data format	*Analyzed*
Experimental factors	*Cytokine levels in the bronchoalveolar lavage fluid, innate and adaptive immune response, daily weights, and viral lung titers were collected to determine the effect of intranasal IFNγ in RSV-infected adult mice.*
Experimental features	*The immune response and viral clearance will be compared in RSV-infected adult mice receiving intranasal IFNγ or PBS.*
Data source location	*Pittsburgh, USA*
Data accessibility	*The data will be available with this article*

**Value of the data**

• The data demonstrates that adult mice infected with RSV have delayed IFNγ production and could be used by others developing immunotherapies for RSV disease.
• The data describes innate and adaptive immune responses to locally delivered IFNγ during adult RSV infection which is frequently targeted by RSV vaccine candidates and may inform vaccine strategies.
• Describes the effect of intranasal cytokine delivery on viral clearance in adult mice and allows other researchers to extend this data to further investigations.
• Demonstrates the safety of intranasal IFNγ delivery in RSV-infected adult mice and provides evidence of potential clinical applications with additional studies.

## 1 Data

**Fig. 1:**
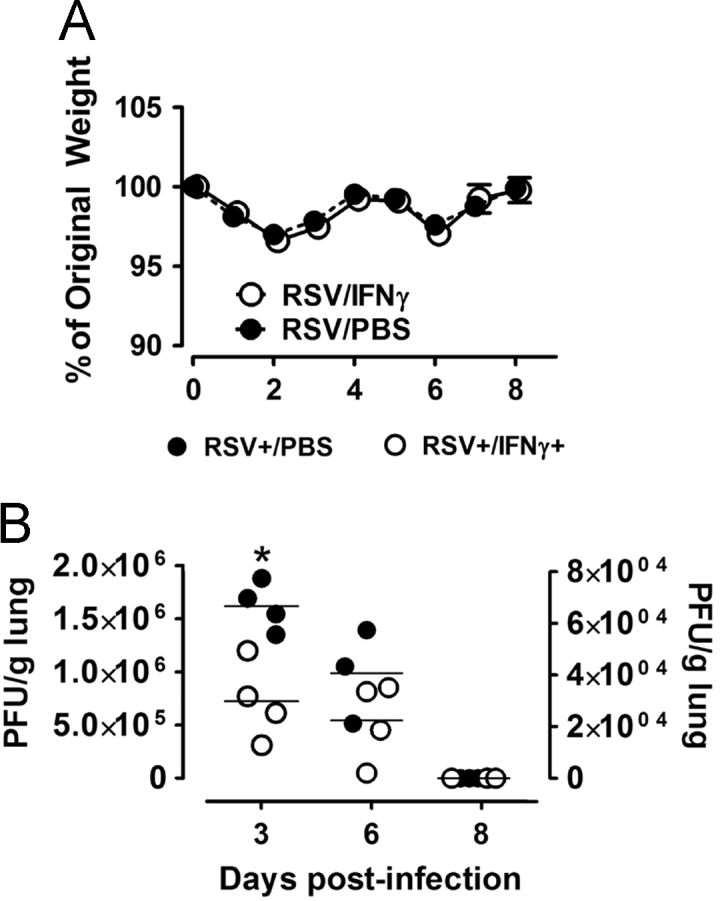
The percent change in daily weights compared to baseline (A) and viral clearance over time (B) are shown in RSV-infected adult BALB/c mice treated with intranasal IFNγ or PBS. A one-way ANOVA indicates significant differences between the groups; *p< 0.05.

**Fig. 2:**
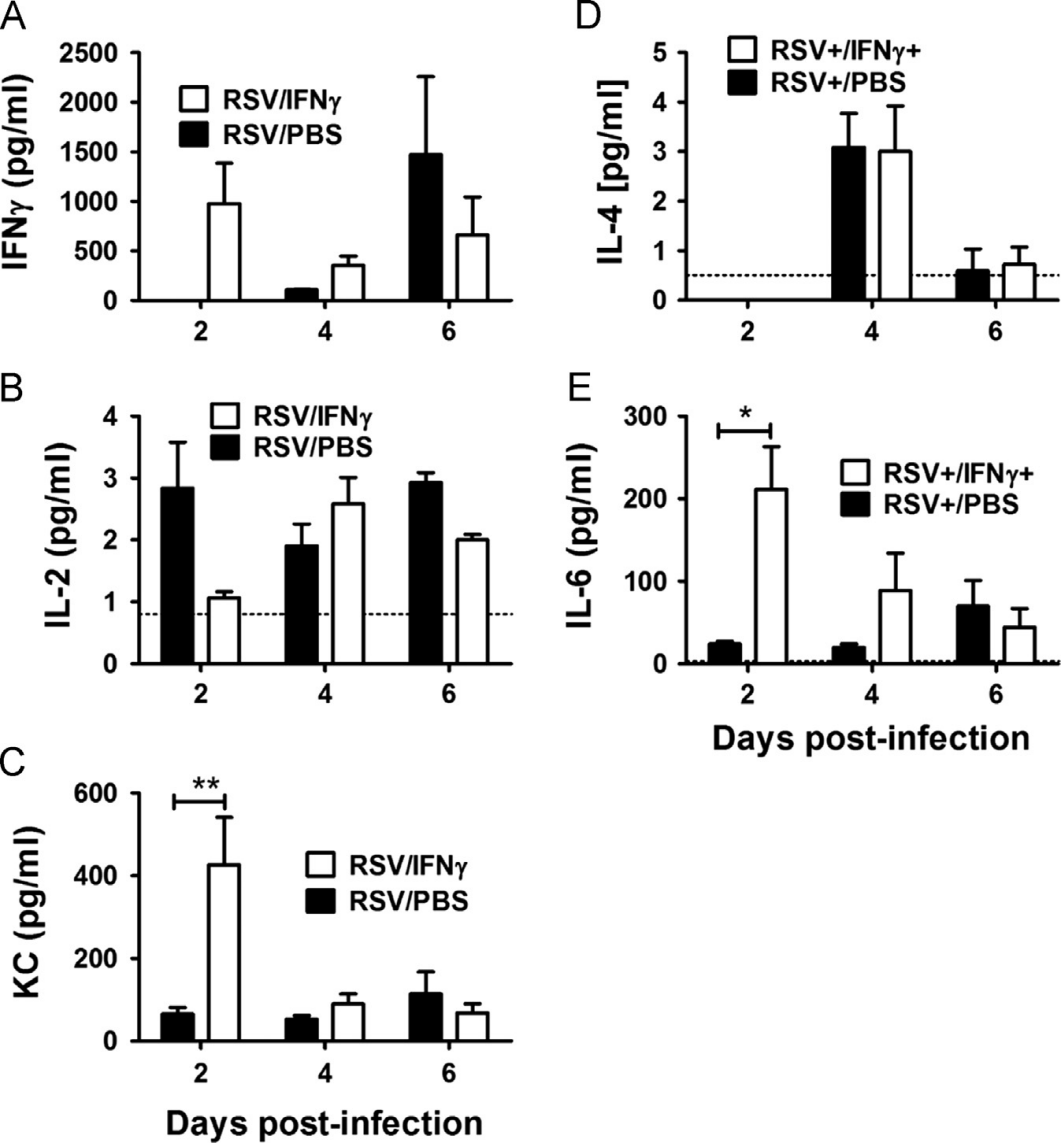
Cytokine production in the BALF (A-E) of adult BALB/c mice infected with RSV are shown for groups treated with intranasal IFNγ or PBS. Mean and SEM are reported with ≥ 5 mice per group; a one-way ANOVA describes group differences: *p<0.05; **p<0.01.

**Fig. 3:**
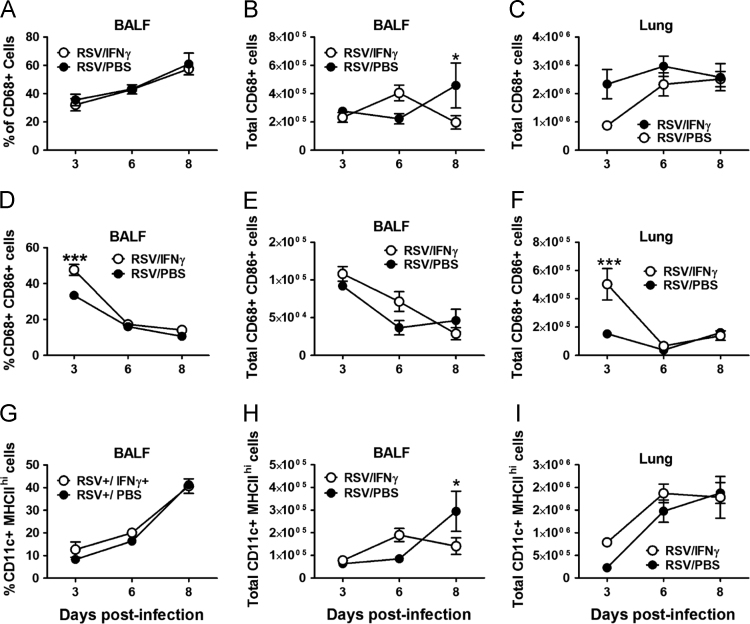
The expression of CD68+ macrophages (A-C), CD86+ CD68+ macrophages (D-F), and CD11c+ MHCIIhi dendritic cells (G-I) are reported as percent of large cells in the BALF (A, D, G), total cells in the BALF (B, E, H), and total cells in digested whole lung tissue (C, F, I) following treatment with IFNγ or PBS. Mean and SEM are reported with ≥ 5 mice per group; a one-way ANOVA describes group differences: *p<0.05; ***p<0.001.

**Fig. 4:**
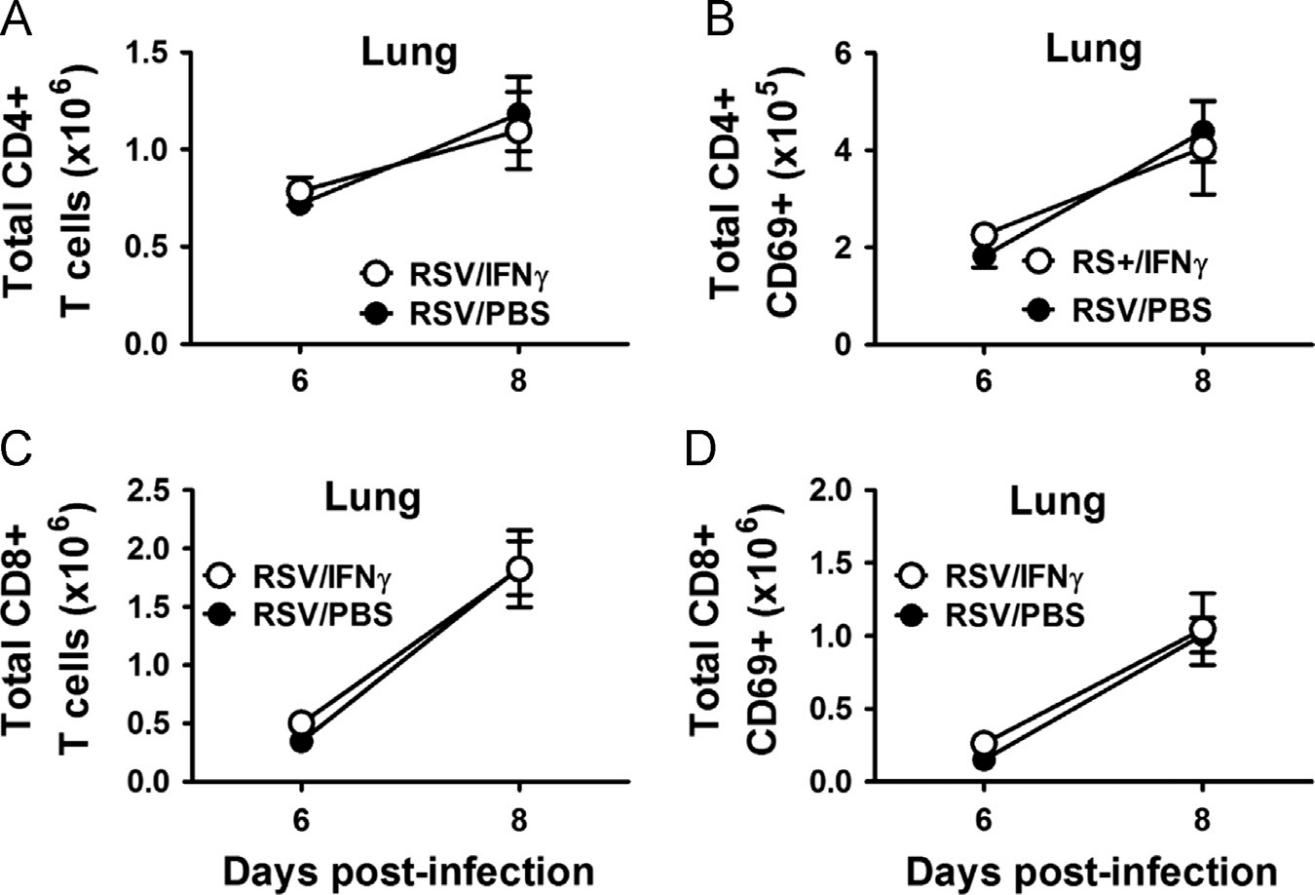
Total CD4 (A) and CD8 (C) T cells and total activated (CD69+) CD4 (B) and CD8 (D) T cells are reported for each group in digested lung tissue (lung) at 6 and 8 dpi in RSV-infected adult mice following treatment with intranasal IFNγ or PBS. No significant differences were determined between groups using a one-way ANOVA.

**Fig. 5:**
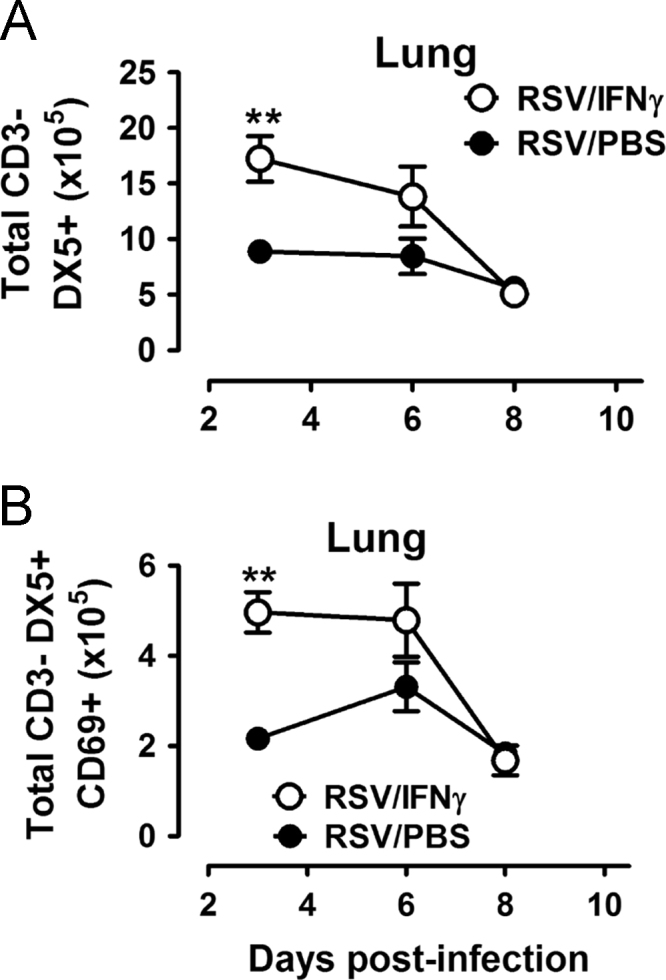
Total DX5+ NK cells (A) and activated (CD69+) NK cells (B) in digested lung tissue (lung) are reported in RSV-infected adult mice treated with intranasal IFNγ or PBS. Mean and SEM are reported with ≥ 5 mice per group; a one-way ANOVA describes group differences: **p<0.01.

The dataset of this article describes delayed IFNγ production in adult mice infected with RSV as well as the immune response and viral clearing effects of IFNγ when delivered intranasally compared to PBS alone. Fig. 1 shows RSV-mediated weight changes and viral clearance in adult mice treated with intranasal IFNγ or PBS. Fig. 2, Fig. 3, Fig. 4, Fig. 5 show changes in innate and adaptive immunity in RSV-infected adult mice treated with IFNγ or PBS.

## 2 Experimental design, materials and methods

Balb/cJ mice aged 6–8 weeks, were ordered from The Jackson Laboratory, Bar Harbor, ME and were maintained in pathogen-free facilities in the Division of Laboratory Animal Resources at the University of Pittsburgh (Pittsburgh, PA). Experiments and animal handling were performed according to protocols approved by The University of Pittsburgh Institutional Animal Care and Use Committee. Where indicated, mice were infected intranasally (i.n.) with RSV Line 19 (RSV L19, Martin Moore, Emory University, Atlanta, GA) (5 × 10^5^ pfu/g, ~1.5 × 10^6^ pfu in 100 μl) under isoflurane anesthesia. On one day post infection (dpi) 50 μl of recombinant murine IFNγ (16 ng/g) (Peprotech, Rocky Hill, NJ) or vehicle only (PBS) were delivered intranasally to RSV-infected mice under light isoflurane anesthesia on 1, 3, and 5 dpi. Mice were weighed daily; percent change from baseline weight was reported. At the indicated times post-infection, at least 5 mice per group were culled for tissue collection. Lungs were lavaged with HBSS-EDTA, then right lungs were harvested and processed for flow cytometry and left lungs were snap frozen for viral plaque assays as previously described [2].
