# Supplementary material for: Data describing IFNγ-mediated viral clearance in an adult mouse model of respiratory syncytial virus (RSV)
Source: Data Brief. 2017 Jul 19;14:272–7. doi: 10.1016/j.dib.2017.07.034 (PMC5567392; doi:10.1016/j.dib.2017.07.034)
Supplement: Supplementary file 1 — Supplementary material [file mmc1.pdf]

University of Pittsburgh

School of Pharmacy
Department of Pharmacy and Therapeutics

207 Salk Pavilion
335 Sutherland Drive
Pittsburgh, Pennsylvania 15261
412-648-9629
E-mail: kme33@pitt.edu

Kerry McGarr Empey, PharmD, PhD
Assistant Professor

June 16, 2017

Dear Editor,

We wish to confirm that there are no known conflicts of interest associated with this publication and there has been no significant financial support for this work that could have influenced its outcome. We confirm that the manuscript has been read and approved by all named authors and that there are no other persons who satisfied the criteria for authorship but are not listed. We further confirm that the order of authors listed in the manuscript has been approved by all of us. We confirm that we have given due consideration to the protection of intellectual property associated with this work and that there are no impediments to publication, including the timing of publication, with respect to intellectual property. In so doing we confirm that we have followed the regulations of our institutions concerning intellectual property. We further confirm that any aspect of the work covered in this manuscript that has involved experimental animals has been conducted with the ethical approval of all relevant bodies and that such approvals are acknowledged within the manuscript.

We understand that the Corresponding Author is the sole contact for the Editorial process (including Editorial Manager and direct communications with the office). He/she is responsible for communicating with the other authors about progress, submissions of revisions and final approval of proofs. We confirm that we have provided a current, correct email address which is accessible by the Corresponding Author and which has been configured to accept email from kme33@pitt.edu.

Kind Regards,

Kerry Empey

Katherine M. Eichinger

Kerry Empey, PharmD, PhD
Assistant Professor
Center for Clinical Pharmaceutical Sciences
Department of Pharmacy and Therapeutics
Pittsburgh School of Pharmacy
Department of Immunology, School of Medicine

Katherine Eichinger, PharmD
Center for Clinical Pharmaceutical Sciences
Department of Pharmacy and Therapeutics
Pittsburgh School of Pharmacy
